# Novel analytical methods to study the fate of mycotoxins during thermal food processing

**DOI:** 10.1007/s00216-019-02101-9

**Published:** 2019-10-21

**Authors:** David Stadler, Franz Berthiller, Michele Suman, Rainer Schuhmacher, Rudolf Krska

**Affiliations:** 1 Institute of Bioanalytics and Agro-Metabolomics, Department of Agrobiotechnology (IFA-Tulln), University of Natural Resources and Life Sciences, Vienna (BOKU), Konrad-Lorenz-Str. 20, 3430 Tulln, Austria; 2 Barilla G. R. F.lli SpA, Advanced Laboratory Research, Via Mantova 166, 43122 Parma, Italy; 3 Institute for Global Food Security, School of Biological Sciences, Queens University Belfast, University Road, Belfast, Northern Ireland BT7 1NN UK

**Keywords:** Food contaminants, Thermal degradation, Trichothecenes, Liquid chromatography, Mass spectrometry

## Abstract

Food processing can lead to a reduction of contaminants, such as mycotoxins. However, for food processing operations where thermal energy is employed, it is often not clear whether a reduction of mycotoxins also results in a mitigation of the toxicological impact. This is often due to the reason that the formed degradation products are not characterized and data on their toxicity is scarce. From the perspective of an analytical chemist, the elucidation of the fate of a contaminant in a complex food matrix is extremely challenging. An overview of the analytical approaches is given here, and the application and limitations are exemplified based on cases that can be found in recent literature. As most studies rely on targeted analysis, it is not clear whether the predetermined set of compounds differs from the degradation products that are actually formed during food processing. Although untargeted analysis allows for the elucidation of the complete spectrum of degradation products, only one such study is available so far. Further pitfalls include insufficient precision, natural contamination with masked forms of mycotoxins and interferences that are caused by the food matrix. One topic that is of paramount importance for both targeted and untargeted approaches is the availability of reference standards to identity and quantity the formed degradation products. Our vision is that more studies need to be published that characterize the formed degradation products, collect data on their toxicity and thereby complete the knowledge about the mycotoxin mitigating effect during food processing.

## Introduction

Food processing is the transformation of agricultural products into food. Unfortunately, many agricultural crops are frequently contaminated with mycotoxins (i.e., low-molecular-weight, secondary metabolites of fungi which are toxic to animals and humans even in low concentrations). Starting roughly 30 years ago, extensive research was carried out to investigate whether food processing can reduce the toxicological impact of mycotoxins [1]. Among the most investigated mycotoxins during food processing are (i) trichothecenes during wheat processing (e.g. milling and baking) [2], (ii) fumonisins and aflatoxins during the production of maize-based products, such as tortillas and cornflakes, [3] and (iii) aflatoxins during sorting of nuts and drying of fruits [1].

Most studies on the fate of mycotoxins during food processing focus on the reduction of the concentration of the parent mycotoxin from the raw material to the finished food product. This approach is suitable for food processing operations that do not cause the degradation of the parent mycotoxin. Among such food processing operations are processes where the concentration of mycotoxins is redistributed between two fractions (e.g. sieving, milling and sorting [1, 4]). However, in food processing operations where thermal energy is used (e.g. baking [5], nixtamalization [3], extrusion [6] and roasting [7]), mycotoxins may be degraded by reactions with chemicals, enzymes or matrix components. The degradation products may have different toxicokinetic and toxicodynamic properties than the parent mycotoxin. Therefore, detailed knowledge about the fate of mycotoxins during food processing is imperative for a complete risk assessment of the consumption of food which was produced from raw materials that are contaminated with mycotoxins. Although the European Food Safety Authority (EFSA) reported that thermal food processing may have an important impact on mycotoxin mitigation, major uncertainties concerning the formed degradation products and their toxicity were acknowledged [8].

To gain complete understanding of a mycotoxins’ behaviour during food thermal food processing, the degradation products have to be identified and accurately quantified. However, we acknowledge from our own experience that this is challenging due to the following reasons:

- The complexity of food matrices can lead to a diverse set of degradation products. Reference standards have to be synthesized to identify and quantify the degradation products.
- The thermal stability of mycotoxins in combination with mild processing conditions that are used during food processing often results in a low amount of a mycotoxin that is degraded. This implies that the analytical methodology needs to be highly accurate and sensitive.
- As the food matrix continuously changes during food processing, the analytical methodology has to be validated for each processing step that is of interest.

The objectives of this article are to (i) provide an overview of the analytical methodologies that can be used to determine the fate and behaviour of mycotoxins during food processing and (ii) to exemplify their applications and limitations.

## Effect of food processing on mycotoxins

As food processing is considered to be an important tool for mycotoxin mitigation, many studies on the efficacy of mycotoxin degradation strategies have been published. Thermal food processing was shown to be effective in reducing the concentration of trichothecenes (e.g. deoxynivalenol (DON)), ochratoxin A (OTA), zearalenone (ZEN), aflatoxins (e.g. aflatoxin B_1_ (AfB_1_)) and fumonisins (e.g. fumonisin B_1_ (FB_1_)) [1]. Although many studies on the reduction of mycotoxins during food processing exist, much less information about the formed degradation products is available. Food processing can lead to the formation of covalent adducts of the parent mycotoxin with matrix components, such as proteins or starch. This was observed for OTA during coffee roasting [9] and is suspected for fumonisins during the production of maize-based products such as cornflakes [10]. Structural modifications of the parent mycotoxin that can occur during food processing include isomerization, decarboxylation, rearrangements and the reaction with other small molecules (Table 1).

**Table 1:** Overview of the degradation products of mycotoxins during thermal food processing. *FB*_*1*_, fumonisin B_1_, *OTA*, ochratoxin A, *DON*, deoxynivalenol, *NIV*, nivalenol, *PHFB*_*1*_, partially hydrolyzed FB_1_, *HFB*_*1*_, hydrolyzed FB_1_, *NDF*, N-(1-deoxy--fructose-1-yl), *NCM*, N-(carboxymethyl)

Food process	Parent mycotoxin	Degradation products
Baking	DON NIV	isoDON, norDONs A–C [5, 11, 18] norNIVs A–C [12]
Nixtamalization	FB_1_	PHFB_1_, HFB_1_, NDF-FB_1_, NCM-FB_1_ [13] FB_1_-polysaccharide, FB_1_-protein [10]
Coffee roasting	OTA	14-R-OTA, 14-decarboxy-OTA [14] OTA-polysaccharide [9]

Toxicity studies show that the degradation products can have a completely different toxicity than the parent mycotoxins. For example, isoDON [5], norDONs A–C [11], norNIVs A–C [12] and NCM-FB_1_ [13] were found to be considerably less toxic than the respective parent mycotoxin. Some degradation products, such as 14-R-OTA which was shown to be a factor 10 less cytotoxic than OTA, can retain some toxicity [14]. Therefore, the identification and quantification of not only the parent mycotoxin but also the formed degradation products are essential for a complete risk assessment of the toxicological impact of mycotoxins that are present in thermally processed food commodities.

## Thermal degradation products of mycotoxins—beyond routine analysis

At present, the knowledge about thermal degradation products of mycotoxins is limited as they are not detected by routine analysis of mycotoxins. Routine detection of mycotoxins is restricted to an a priori selected set of target analytes. Currently, this set of analytes often consists of mycotoxins that pose a public health concern and which are therefore regulated in certain commodities [15]. Therefore, the analytical chemist has to develop appropriate analytical approaches for the identification and quantification of the thermal degradation products of mycotoxins in various food matrices.

**Fig. 1:**
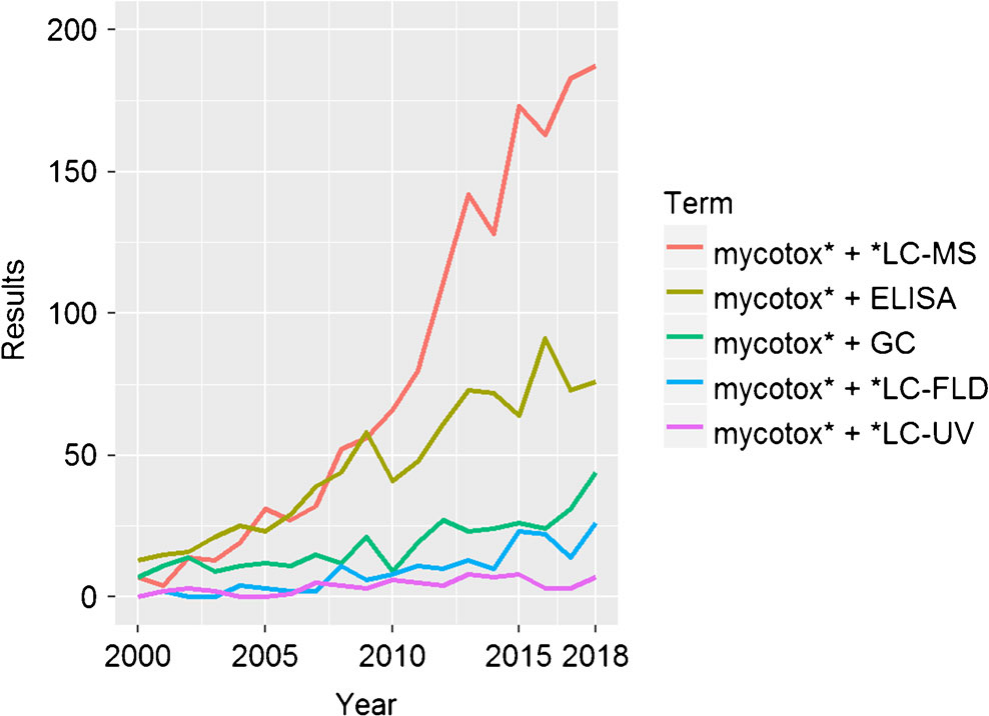
Number of publications found in Web of Science (https://apps.webofknowledge.com, accessed 11.04.2019)

When reviewing the analytical techniques that are used in mycotoxin research, it becomes evident that especially in the last decade there is an obvious trend towards liquid chromatography-mass spectrometry (LC-MS)–based analysis (Fig. 1).

It is therefore no surprise that recent studies on mycotoxin degradation during food processing rely almost exclusively on LC-MS-based analysis. Although LC-MS is an extremely helpful tool for detecting compounds in trace-level amount in complex matrices, there are pitfalls for the identification and quantitation of mycotoxins that should be avoided. Therefore, the different approaches and their limitations for the identification and quantification of thermal degradation products will be discussed in detail.

## Identification of the degradation products

The degradation products that are formed during food processing can be elucidated by targeted or untargeted analysis. The features of these two approaches are summarized in Table 2. The targeted approach requires reference standards for the identification and quantification of the analytes. Putative analyte characterization as well as relative quantitation can be carried out using the untargeted approach. However, for the identification of the compounds reference standards need to be available as well.

**Table 2:** Definition and comparison of the untargeted and targeted approach for the determination of the fate of mycotoxins during food processing

	Targeted	Untargeted
Definition [8]	Any analytical method that is aimed at the determination of a specific analyte or of a group of analytes. The analytical procedure should be properly validated for the target “known” analytes	An analytical method, mainly based on mass spectrometry followed by data mining and elaboration, aimed at the acquisition of undefined information from a sample (“profiling”). Information about “known” and “unknown” analytes can be obtained in the post-acquisition data elaboration
Analyte identification [27]	Identification • Authentic reference standards	Unknown compounds • No information is available Putatively characterization • Use certain properties that indicate analyte class Putatively annotation • Similarities in physiochemical properties and/or spectral similarities with spectral libraries Identification • Authentic reference standards
Quantification	Absolute	Relative
Advantages	• Identification and accurate quantitation of a defined set of analytes which is predetermined by the scientific question at hand • Low limit of detection/quantification can be achieved	Find the complete spectrum of the extractable degradation products
Disadvantages	Analytes that are formed and not part of the defined set of analytes are not discovered	Without authentic reference standards, analyte quantitation is not possible. • Often the majority of the analytes cannot be characterized, annotated and/or identified and remains “known unknowns”

### Targeted approaches

Contrary to mycotoxins that have been of greater interest in the scientific community such as regulated, masked and emerging mycotoxins [16, 17], no reference standards are commercially available for thermal degradation products that are formed during food processing. Therefore, in previous studies that used a targeted approach, a set of reference standards was synthesized (Table 3). Reference standards were obtained by degradation of a standard in either pure solvent or in a solution containing compounds that imitate the food matrix. The degradation products were then isolated using preparative chromatography and their structure was elucidated by nuclear magnetic resonance spectroscopy (NMR) [5, 11, 12, 18]. A targeted search was then conducted for these compounds in food samples that were produced from naturally contaminated raw materials.

**Table 3:** Comparison of the thermal degradation products of mycotoxins found in model experiments and that confirmed in food matrices. *FB*_*1*_, fumonisin B_1_, *OTA*, ochratoxin A, *DON*, deoxynivalenol, *NIV*, nivalenol, *PHFB*_*1*_, partially hydrolyzed FB_1_, *HFB*_*1*_, hydrolyzed FB_1_, *NDF*, N-(1-deoxy-d-fructose-1-yl), *NCM*, N-(carboxymethyl)

Parent mycotoxin	Degradation products	
	Solvent or model experiments	Confirmed in food samples
DON	isoDON, norDONs A–F, DON-lactone, 9-hydroxymethyl DON lactone [11, 18]	isoDON, norDONs A–C [5, 11, 18]
NIV	norNIVs A–C, NIV lactone [12]	norNIV B [12]
T-2 toxin	Compounds 1–3 [21]	Compound 3 [21]
FB_1_	PHFB_1_, HFB_1_, NDF-FB_1_, NCM-FB_1_, FB_1_-polysaccharide, FB_1_-protein [10, 33, 34]	PHFB_1_, HFB_1_, NCM-FB_1_, NCM-FB_1_, FB1-protein [34, 35]
OTA	14-R-OTA, 14-decarboxy-OTA, OTA-α-amide, OTA-polysaccharide [9, 14]	14-R-OTA, 14-decarboxy-OTA OTA-polysaccharide [9, 14]

However, some of the degradation products that can be formed during food processing can also be present in the raw materials. This was observed for isoDON and norDONs B–C that were found as natural contamination of flour [5]. Therefore, by analyzing food items for these degradation products without knowing their concentration in the raw materials might lead to flawed results.

**Fig. 2:**
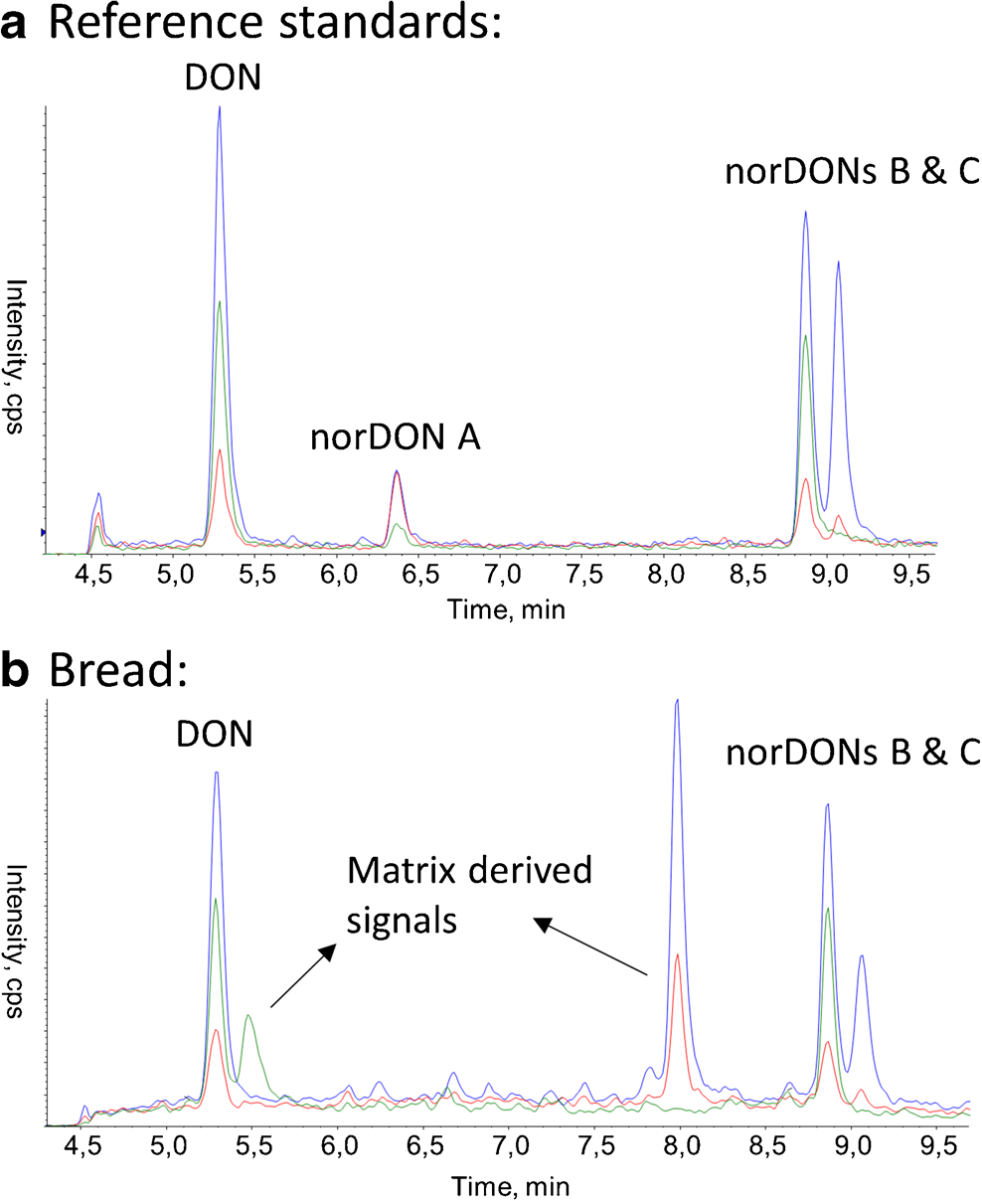
Liquid chromatography tandem mass spectrometry chromatogram of mixture of standards of neat solvent (top) and a bread sample made from dough which was fortified with deoxynivalenol (DON) [5]. Different colors represent different mass transitions

Although laborious in their production, reference standards are essential for the identification of thermal degradation products of mycotoxins. Structural similarities between the parent mycotoxins or the degradation products can lead to chromatographic peaks with similar retention behaviour and similar fragmentation pattern. The detection of an analyte using selected reaction monitoring (SRM) is highly selective. However, signals derived from matrix compounds present in complex food matrices can have the same SRM transitions as mycotoxin degradation products. For example, without reference standards, matrix-derived signals could have been mistaken for degradation products of DON that are formed during the production of bread (Fig. 2).

In some studies, the increase of an analyte is used to justify the conclusion that it is a mycotoxin degradation product. In this way, DOM-1 was falsely concluded to be a degradation product of DON during baking [19]. Generally, in such cases, the increase of an analyte needs to be statistically significant. Since signal suppression/enhancement and recovery factors may even vary for different batches of the same matrix, the statistical significance of the result must be accompanied by a careful evaluation of the measurement uncertainty [20].

The biggest limitation of targeted approaches is that the predetermined set of compounds may differ from the degradation products that are actually formed during food processing. For the trichothecenes DON, NIV and T2-toxin, degradation products that were formed in model experiments were not found in food matrices (Table 3). On the other hand, the complexity of the food matrix may lead to the formation of degradation products that are not within the set of the predetermined targeted analytes. This was observed in a study on the fate of T2 during the production of biscuits [7]. Although the T2 concentration was reduced by 45%, none of the degradation products of a T2 standard (i.e. compounds 1–3 [21]) was found in the finished biscuits. Therefore, by using a targeted approach, it is uncertain whether all relevant degradation products of mycotoxins that are formed in complex food matrices can be found.

### Untargeted approaches

Untargeted LC-high-resolution mass spectrometry (HRMS)–based approaches aim at the unbiased measurement of all low-molecular-weight constituents of biological samples and are therefore in principle suited to solve the abovementioned challenge. In a very recent study, the two open-source software packages MZmine 2 [22] and XCMS online [23] have been used to search for putative derivatives of the mycotoxin T-2 toxin and HT-2 toxin during heating in a simulated food environment [24]. For highly complex matrices like (processed) food, however, these approaches and tools are not well suited since the search for degradation products by simple differential screening typically results in a large number of putative candidates, which are very difficult to verify and interpret.

**Fig. 3:**
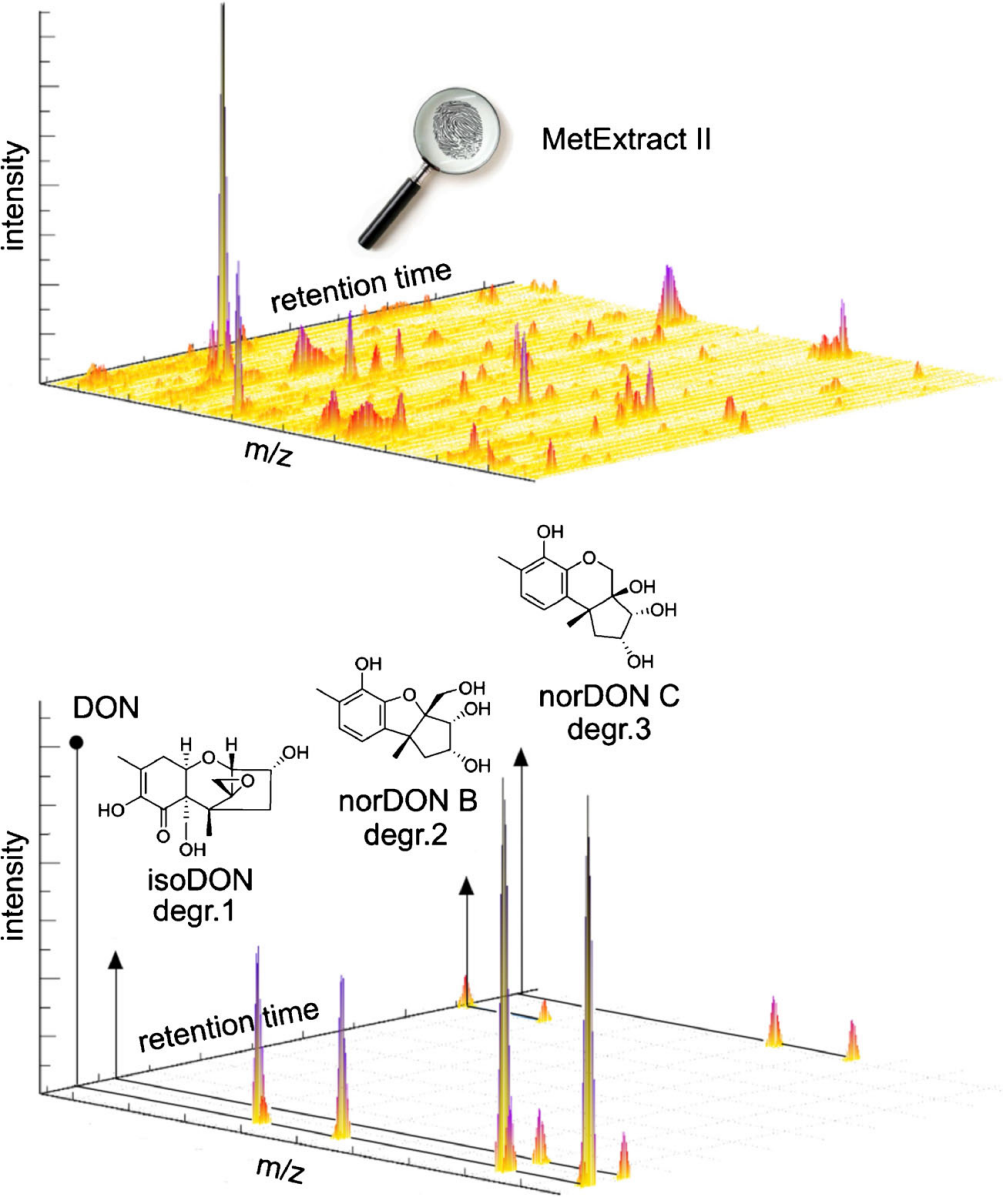
Schematic representation of the untargeted search for the degradation products of deoxynivalenol (DON) that are formed during the production of bakery products [5]. Top: High-resolution liquid chromatography high-resolution mass spectrum of a bakery product that was prepared from dough that was fortified with DON and ^13^C-labelled DON. Bottom: Signals that carried the unique isotopic fingerprint were filtered from the mass spectrum using the software tool MetExtract II. The identification of the putatively annotated degradation products (degr.1–3) was carried out by comparison with reference standards

In contrast, the use of stable isotopically labelled tracer compounds in combination with LC-HRMS offers great potential for the untargeted screening of mycotoxin degradation products in complex food and feed samples. An isotope-assisted method that was originally been developed for untargeted metabolomics studies was adapted to study the fate of DON in wheat [25, 26] as well as of T-2 and HT-2 toxins in oats, wheat and barley [27–29]. Recently, the method was also used to study the fate of DON during industrial baking [5]. In brief, bakery products were prepared from a dough that was fortified with DON and ^13^C-labelled DON. This results in a unique isotopic fingerprint that is carried by all of the formed degradation products. The software tool MetExtract II [30] was used to filter the signals of the degradation products from the LC-HRMS spectrum of a bakery product (Fig. 3). Reference standards were used to confirm the identity of the formed degradation products.

Using this untargeted approach, all known and (potentially) unknown degradation products of a tracer can be determined in an unbiased way. However, the Achilles heel of many metabolomics studies is the identification of the annotated compounds, as reference standards often are not available [29, 31]. Therefore, the synthesis and characterization of reference standards are essential to identify putatively annotated degradation products.

## Accurate quantification

Contrary to the identification of the degradation products, absolute quantitation of mycotoxins is exclusively carried out in a targeted manner. Due to the reasons mentioned in the introduction, absolute quantitation of the degradation of mycotoxins in food matrices is challenging and requires highly accurate and sensitive methods.

Upon reviewing literature reports which analyzed the effect of food processing mycotoxins, we found issues relating to the accuracy of LC-MS-based analysis that are worth considering before executing the experimental trials.

**Fig. 4:**
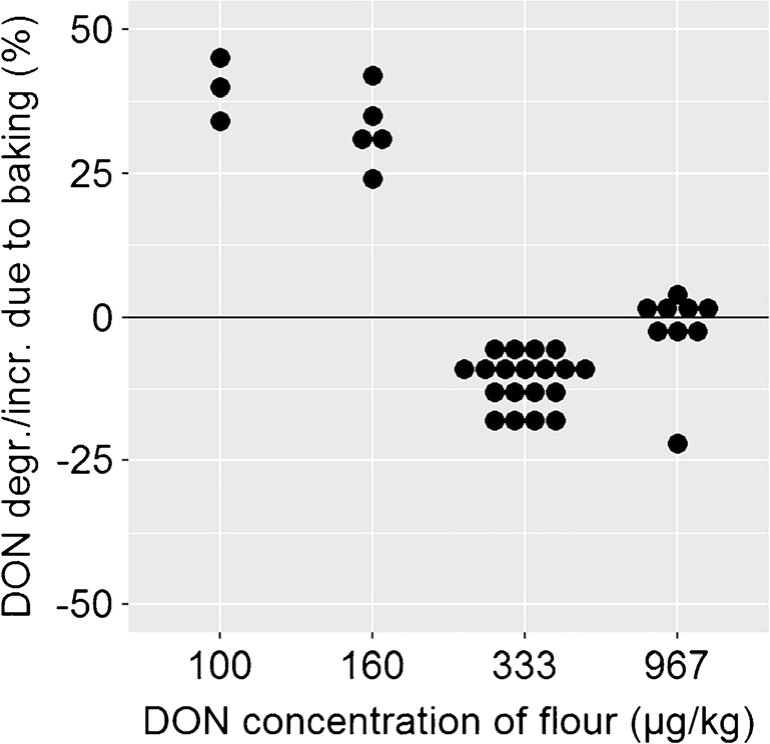
Deoxynivalenol (DON) degradation/increase that was found during the production of bread from batches of flour with a varying DON concentration [28]

The first issue concerns the potential bias that can arise from the experimental setup and the presence of masked mycotoxins. Masked mycotoxins are plant metabolites of mycotoxins (e.g. DON-3-glucoside) that are often not detected in routine analysis [17]. In naturally contaminated raw materials, masked mycotoxins often co-occur with the mycotoxin of interest. During food processing, these masked forms might be partly converted to the parent mycotoxin and thereby causing a biased result. This was observed when bread was baked from four batches of flour (Fig. 4) [32].

For the two batches of flour which were contaminated with a low concentration of DON, an increase of DON during baking was observed. From bread produced from the two highly contaminated batches of flour, a degradation of DON was observed. Therefore, Bergamini et al. hypothesized that the release of masked mycotoxins leads to an apparent increase of the DON concentration during bread baking. Since neither the starting material nor the baking products were analyzed for masked forms of the mycotoxin of interest, no sound conclusion about the extent of degradation can be made from this study [32]. By characterizing the starting material for masked forms of mycotoxins, such potential biases can be avoided.

The determination of mycotoxins that are bound to macromolecules (e.g. fumonisins) is particularly difficult. The formation of a covalent bond between mycotoxins and matrix constituents renders them inaccessible to conventional solvent extraction. Such bound fumonisins can be released from the matrix under alkaline conditions. In this way, matrix-associated fumonisins were found to be present at similar or even higher amounts than the free forms in maize and various corn-based products [33, 34]. Evidence of the formation of matrix-bound forms of mycotoxins during food processing was detected by heating a mixture of a mycotoxin and a substance modelling certain food constituent (e.g. polysaccharides). Using this strategy, matrix-associated forms of OTA that are formed during coffee roasting have been described [9]. For the quantitation of the matrix-bound OTA, a sample preparation protocol using enzymatic cleavage of the matrix had to be used. However, absolute quantitation of matrix-bound forms of mycotoxins remains challenging as special procedures for the cleavage of the mycotoxins-matrix bond have to be developed and validated.

As in some studies the experiments are not precisely described, it is often unclear whether or not the data were corrected for the recovery of the analytes and the change in moisture content [2]. In such cases, it is impossible to judge from an outside perspective whether biases that could arise from incomplete recovery of an analyte, changes in moisture content or dilution with non-contaminated ingredients were taken into account.

**Fig. 5:**
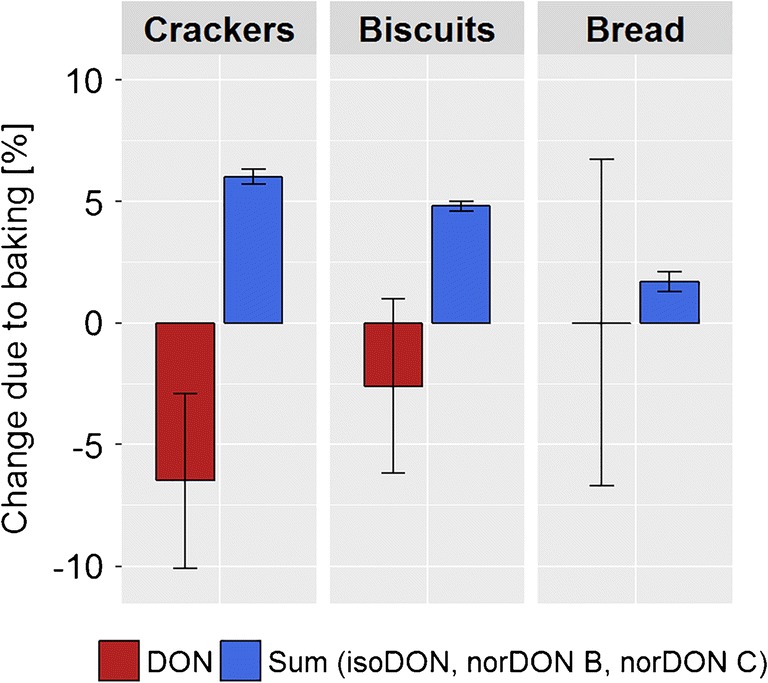
Comparison of the accuracy for the quantitation of the degradation of the parent mycotoxin and the increase of its degradation products [5]. Error bars represent the standard deviation

In addition to potential biases, the precision of a method is an important criterion. To get an estimate of the precision of state-of-the-art methods for the quantification of mycotoxins in food matrices, we compared the relative standard deviation (RSD) of DON concentration values in bread (Fig. 5) [5, 32].

For both methods, the precision as measured by the RSD of the results was in the range of 5–10%. For the determination of trace levels of a chemical in a complex matrix, these methods can be considered highly precise. However, the precision may not be sufficient for the quantification of the minor mycotoxin degradation products formed during food processing. Especially in the case of bread, where due to the low surface ratio the degradation of DON ranges between 2 and 5% only, the precision of state-of-the-art analytical methodology is not sufficient to accurately determine the DON degradation [5, 35]. This seems to be the major reason for the inconsistent degree of DON degradation during baking as reported in the literature [2, 36].

Obtaining the first complete mass balance of the degradation of mycotoxins and the increase of the degradation products revealed that the uncertainty associated with the DON degradation was considerably higher than the uncertainty associated with the increase of the degradation products (Fig. 5) [5]. Since the increase of the degradation products could be determined much more accurately, we consider the increase of the degradation products a much more accurate measure for DON degradation than the decrease of the DON concentration.

## Outlook—vision

Information in research articles on the fate of mycotoxins during food processing is mostly restricted to the effect of a processing method on the concentration of the parent mycotoxin. In this way, information on the formed degradation products is not collected. However, the toxicity of the formed degradation products is essential to make a sound conclusion on the potential impact of the baking process and the parameters chosen on the safety of the final food product. Only when a decrease of a mycotoxin results in the formation of degradation products that are less toxic than the parent mycotoxin, food processing can be considered to have a mycotoxin mitigating effect.

Therefore, priority should be given to the identification and isolation of the formed degradation products and the study of their toxicity. Only when the fate of a mycotoxin is clarified, further experiments on the amount the mycotoxin that can be degraded under certain processing conditions should be set up.

One topic that is of paramount importance for both targeted and untargeted approaches is the availability of reference standards. We are of the firm opinion that the extra effort that is necessary for obtaining reference standards pays off in terms of erasing any doubt concerning the identity, quantity and toxicity of a formed degradation product and will lead to a substantially higher quality of the generated data.

A general trend that is seen in recently published multi-mycotoxin methods is the increased use of high-resolution mass spectrometry, in particular by using (quadrupole-)time-of-flight or orbitrap-based mass spectrometers. One major advantage of those measurements is the possible retrospective data analysis. Studies conducted only focusing on the native mycotoxin might later be revisited, once degradation products are described. Using accurate mass measurements, the identity of degradation products can be confirmed later by running authentic reference standards and comparing retention times. While accurate post-acquisition quantification is impossible, comparing peak heights with standards can still yield semi-quantitative information about the extent of degradation.

High-resolution mass spectrometry is almost imperative for structural elucidation of formed mycotoxin degradation products, unless standards were produced previously which were characterized by different analytical techniques such as NMR. The intrinsic difficulty of finding the needle in the haystack in interpreting high-resolution MS data can be elegantly overcome by using stable isotope–labelled tracers. Such (uniformly) labelled compounds became much more available in the last 10 years and are mainly used to control matrix effects in mass spectrometry. With the larger availability of both labelled compounds and HRMS instruments, we envisage a rise in the determination of the fate of food constituents and contaminants during food processing.

So far, studies focusing on the elucidation of the degradation products are only available for a limited number of mycotoxins in certain food processing operations. These studies show the enormous efforts necessary to identify the degradation products and characterize their toxicity. Our vision is that the current knowledge about the fate of mycotoxins and other contaminants during food processing will be expanded to other food processing operations. In this way, food producing companies and risk assessors will be provided with valuable data that make an important contribution to the safety of our food items.
